# Active nNOS Is Required for Grp94-Induced Antioxidant Cytoprotection: A Lesson from Myogenic to Cancer Cells

**DOI:** 10.3390/ijms23062915

**Published:** 2022-03-08

**Authors:** Filippo Fornasiero, Cristina Scapin, Maurizio Vitadello, Paola Pizzo, Luisa Gorza

**Affiliations:** 1 Department of Biomedical Sciences, University of Padova, 35131 Padova, Italy; fornasierofilippo1@gmail.com (F.F.); labgorza93@gmail.com (C.S.); paola.pizzo@unipd.it (P.P.); 2 CNR-Neuroscience Institute, National Research Council, 35131 Padova, Italy; maurizio.vitadello@gmail.com

**Keywords:** oxidative stress, Grp94, gp96, nNOS, PMCA, SERCA2, calcium, endoplasmic reticulum, C2C12 cell line, MDA-MB-231 cell line

## Abstract

The endoplasmic reticulum (ER) chaperone Grp94/gp96 appears to be involved in cytoprotection without being required for cell survival. This study compared the effects of Grp94 protein levels on Ca^2+^ homeostasis, antioxidant cytoprotection and protein–protein interactions between two widely studied cell lines, the myogenic C2C12 and the epithelial HeLa, and two breast cancer cell lines, MDA-MB-231 and HS578T. In myogenic cells, but not in HeLa, Grp94 overexpression exerted cytoprotection by reducing ER Ca^2+^ storage, due to an inhibitory effect on SERCA2. In C2C12 cells, but not in HeLa, Grp94 co-immunoprecipitated with non-client proteins, such as nNOS, SERCA2 and PMCA, which co-fractionated by sucrose gradient centrifugation in a distinct, medium density, ER vesicular compartment. Active nNOS was also required for Grp94-induced cytoprotection, since its inhibition by L-NNA disrupted the co-immunoprecipitation and co-fractionation of Grp94 with nNOS and SERCA2, and increased apoptosis. Comparably, only the breast cancer cell line MDA-MB-231, which showed Grp94 co-immunoprecipitation with nNOS, SERCA2 and PMCA, increased oxidant-induced apoptosis after nNOS inhibition or Grp94 silencing. These results identify the Grp94-driven multiprotein complex, including active nNOS as mechanistically involved in antioxidant cytoprotection by means of nNOS activity and improved Ca^2+^ homeostasis.

## 1. Introduction

The endoplasmic reticulum (ER) chaperone Grp94/gp96 displays several selective features, despite its almost ubiquitous and constitutive expression. In addition to being required for the folding of selected client proteins, it interacts with several non-client ones involved in tissue-specific functions [1]. Both features explain the crucial role played by Grp94 at early embryonic development (i.e., the lethal phenotype of Grp94 knock out (KO) blastocysts [2,3]) and in tissue-specific differentiation (i.e., lymphocytes, [4]) and growth (i.e., skeletal muscle [5,6,7]).

Moreover, Grp94 involvement in cell survival and cytoprotection appears pleomorphic. Grp94 is required for survival of muscle progenitors [2] and neuronal cell lines [8], and for cytoprotection of myogenic cells against oxidative stress [9,10], similarly to other ER chaperones [11]. Conversely, it appears dispensable for survival of lymphocytes [12] and breast cancer cells [13], despite its high protein levels in the latter cell types [14]. However, in HER2-overexpressing breast cancer cells, the use of Grp94-specific inhibitors strongly affected cell viability [15], suggesting that Grp94 involvement in cytoprotection reflects cell- or tissue-specific mechanisms.

Our previous investigations with the myogenic cell line C2C12, which was established from primary cultures of regenerating murine thigh muscle [16], indicated that Grp94 expression levels show a negative correlation with the amount of Ca^2+^ released from intracellular stores upon stimulation [9,10], resulting in cytoprotection after exposure to hydrogen peroxide, which increases cytosolic Ca^2+^ load. Like other ER chaperones, Grp94 binds Ca^2+^ and participates in Ca^2+^ homeostasis [8,9,10]. However, it is not known whether Grp94 modulates Ca^2+^ homeostasis directly or through non-client protein interactions. Indeed, Grp94, like other chaperones, displays several Ca^2+^ binding sites [17], which would potentially influence free Ca^2+^ levels within the ER, although the calculated affinity of these sites is rather low considering the actual Ca^2+^ concentration within the organelle.

The goal of the present study was to clarify the mechanism(s) involved in Grp94-induced antioxidant cytoprotection. To this purpose, we extended our observations from myogenic cells to a non-myogenic cell line, such as HeLa, which is widely used in cell biology research, despite its cancer nature, and investigated the effects of Grp94 overexpression on Ca^2+^ homeostasis and protein–protein interactions. Our results show that the level of expression of Grp94 does not obligatorily reflect the level of cytoprotection but requires the presence of Grp94 interactions with Ca^2+^ handling proteins and nNOS activity. Since we could not validate the results obtained with HeLa cells in non-cancerous primary epithelial cells, we wondered whether the lack of such a Grp94-based cytoprotective machinery characterized the cancer cell phenotype. Therefore, two different breast cancer cell lines, largely used in oncologic research, were also compared for Grp94 expression and involvement in antioxidant cytoprotection and interaction with Ca^2+^ handling proteins and nNOS.

## 2. Results

### 2.1. Effects of Grp94/gp96 Overexpression on Ca^2+^ Homeostasis and Antioxidant Protection in Myogenic and Epithelial Cell Lines

**Figure 1:**
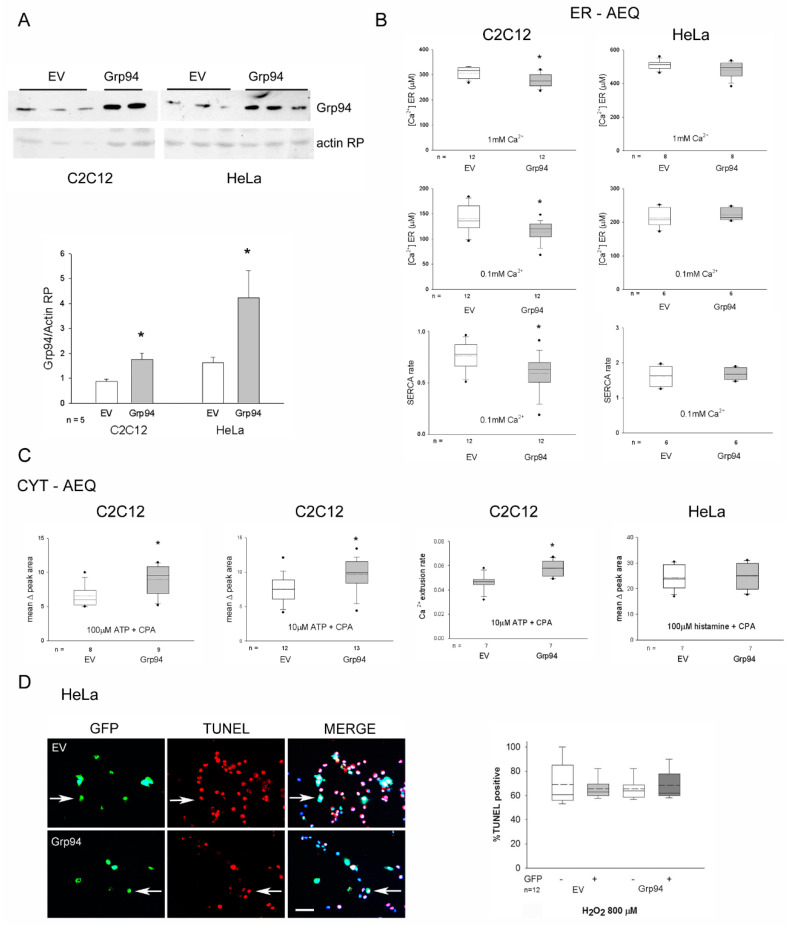
**Grp94/gp96 overexpression differently affects Ca^2+^-homeostasis and antioxidant protection in C2C12 and HeLa cells.** (**A**) Representative Western blots showing Grp94 immunoreactivity in whole homogenates from transiently transfected cultures with empty vector (EV) or Grp94 cDNA. (**B**) Values of ER Ca^2+^ levels determined on EV and Grp94 overexpressing cultures (C2C12 and HeLa) after co-transfection with the ER-AEQ construct and exposure to 1 mM and 0.1 mM Ca^2+^ (upper and middle panels). Lower panels represent rate of SERCA activity, calculated in the presence of Ca^2+^ 0.1 mM, as described in the Methods section. * *p* ≤ 0.02. (**C**) Values of average peak area of cytosolic Ca^2+^ rise (left and right panels) and Ca^2+^ extrusion rate (middle), determined on EV and Grp94 overexpressing cultures (C2C12 and HeLa), after co-transfection with the CYT-AEQ construct and exposure to 100 or 10 µM ATP, or histamine, plus 20 µM CPA in the absence of extracellular Ca^2+^. * *p* ≤ 0.03. (**D**) Left panel: Representative immunofluorescence images of HeLa cells transfected with EV or Grp94 cDNA (green fluorescence due to GFP expression) analyzed for the presence of apoptosis by means of TUNEL (red fluorescence) after exposure to 800 µM hydrogen peroxide for 5 h. Arrows indicate transfected apoptotic cells. Bar: 30 µm. Right panel: Box plots of the percentage of TUNEL positive nuclei calculated on the total amount of GFP-positive cells (about 100 cells for each slide); percentages were also calculated for non-transfected cells (about 300 cells for each slide). n indicates the number of cultures examined from at least two different experiments.

We previously suggested an inverse correlation between the antioxidant protection induced by Grp94 overexpression in C2C12 myogenic cells and the amount of releasable Ca^2+^ from intracellular stores [9,10]. New investigations were then performed using aequorin Ca^2+^ probes, expressed either in the cytosol (CYT-AEQ) or selectively targeted to the ER (ER-AEQ), by means of transient co-transfection with Grp94 cDNA (Grp94) or empty vector (EV) of C2C12 and HeLa cells (Figure 1A).

In Grp94-overexpressing C2C12 cells, ER Ca^2+^ levels at plateau, reached after ER refilling both with 1 mM and 0.1 mM CaCl_2_, appeared significantly reduced compared to those found in control cells (*p* < 0.02; Figure 1B, left). The kinetics of ER Ca^2+^ refilling with 0.1 mM CaCl_2_ was also significantly delayed (*p* < 0.01), suggesting a specific effect of Grp94 on SERCA activity. Despite lower steady-state ER Ca^2+^ levels, cytosolic Ca^2+^ rises induced by ATP (10 or 100 µM), in the absence of extracellular Ca^2+^ and in the presence of the SERCA inhibitor cyclopiazonic acid (CPA), was 20 or 50% higher, respectively (*p* < 0.02), in Grp94-overexpressing C2C12 cells compared to controls (Figure 1C, left). Cytosolic Ca^2+^ rise kinetics showed a significant increase in the rate of recovering basal Ca^2+^ levels in Grp94 overexpressing cells, suggesting an increased PMCA activity (*p* = 0.01; Figure 1C, middle).

Differently from C2C12 cells, Grp94 overexpression in HeLa cells displayed no effect on both ER Ca^2+^ levels and cytosolic Ca^2+^ rises induced by cell stimulation (Figure 1B,C, right), suggesting the lack of Grp94 effect on these parameters. HeLa cells were thus exposed to hydrogen peroxide to evaluate the presence of Grp94-induced cytoprotection. Transfected cells were identified as positive for GFP fluorescence and apoptosis was demonstrated by TdT-mediated dUTP Nick End Labeling (TUNEL) assay using rhodamine-labeled nucleotides (Figure 1D). Percentage of apoptotic HeLa cells did not vary among not transfected and EV- and Grp94-transfected cells, as opposed to murine myogenic cells [10].

### 2.2. Grp94 Interacts with Ca^2+^-Handling Proteins and nNOS

**Figure 2:**
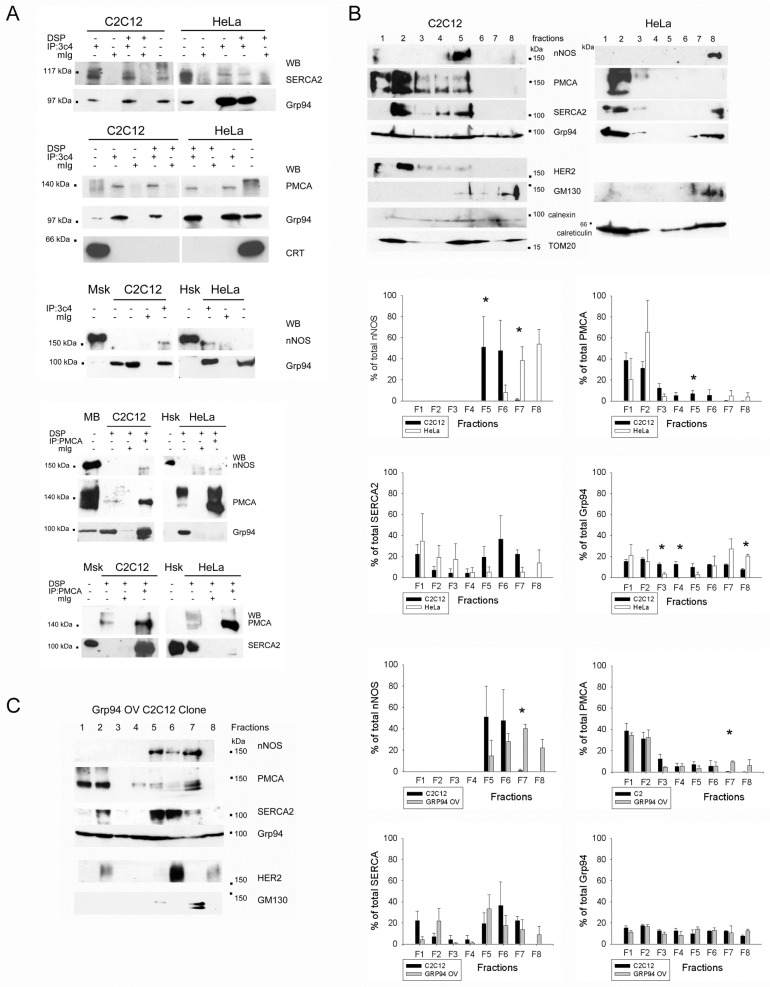
**Grp94 interaction with Ca^2+^ handling proteins and nNOS.** (**A**) Representative Western blots of total lysates (about 30 µg) and immunoprecipitates (IP) from C2C12 and HeLa cultures using anti-Grp94 3c4 antibody (upper panel) or anti-PMCA. Non-immune mouse G immunoglobulins (mIg) were used for control IP. DSP indicates culture exposure to chemical cross-linking before lysis and IP. About 10 µg of mouse diaphragm (Msk) or brain (MB) or human skeletal muscle (Hsk) homogenates were used as a reference for nNOS migration. (**B**) Representative Western blots (upper panels) and histograms (lower panels) of sucrose gradient fractionation of C2C12 and HeLa cultures. HER2 immunoreactivity was used as a plasma membrane marker; GM130 for Golgi; calnexin and calreticulin for ER and TOM20 for mitochondria. Histograms show the distribution of nNOS, PMCA, SERCA2 and Grp94 between C2C12 (black bar) and HeLa (white bar) cells. Values correspond to mean and SEM of 3 different experiments. * *p* ≤ 0.01. (**C**) Representative Western blots (left panel) and histograms (right panels) of sucrose gradient fractionation of a stably Grp94-overexpressing C2C12 clone. HER2 immunoreactivity was used as a plasma membrane marker and GM130 for Golgi. Histograms show the distribution of nNOS, PMCA, SERCA2 and Grp94 between C2C12 cells (black bar) and Grp94-overexpressing ones (grey bars). Values correspond to mean and SEM of 3 different experiments. * *p* < 0.001.

The possibility that the Grp94-dependent defective Ca^2+^ homeostasis and cytoprotection in C2C12 cells derived from specific interactions of the chaperone with non-client proteins was explored by means of immunoprecipitation (IP) studies, using both total and isotype-specific non-immune IgG (Figure 2A and Figure S1). We found that Grp94 co-immunoprecipitated with SERCA2 and PMCA in C2C12 cell lysates (Figure 2A). A similar result was achieved in HeLa cells, where, however, only very low amounts of SERCA2 co-immunoprecipitated, despite the abundancy of this protein in whole lysates. To exclude unspecific interactions with Grp94 or protein involvement in the chaperone machinery, we repeated IP experiments in the presence of the chemical cross-linker DSP. No signal for calreticulin was detected in Grp94 immunoprecipitates from C2C12 and HeLa cells, suggesting that Grp94 interactions did not occur during protein folding (Figure 2A). Since in C2C12 cells Grp94 co-immunoprecipitates nNOS [7], and PMCA can bind nNOS [18], Grp94 IP samples from HeLa cells were investigated and found positive for the presence of nNOS (Figure 2A). Reciprocal IP assays using anti-PMCA antibodies in chemically cross-linked cell homogenates confirmed nNOS, SERCA2 and Grp94 co-immunoprecipitation only in C2C12 cells, while, in HeLa lysates, PMCA did not co-immunoprecipitate with Grp94 and nNOS and SERCA2 retrieved a background level signal only (Figure 2A).

In order to determine whether these interactions took place in the same subcellular compartment, lysates from C2C12 and HeLa cells underwent sucrose discontinuous gradient separation, using a protocol developed to monitor protein distribution among ER vesicles [19]. Different fractions were then tested for nNOS, PMCA, SERCA2 and Grp94 immunoreactivity. As shown in Figure 2B, light, buoyant fractions (F1-3) contained plasma membrane proteins, such as the HER-2 receptor, whereas heavy, non-buoyant, fractions (F7-8) were positive for the Golgi marker GM130. The mitochondrial marker TOM20 appeared enriched in fractions F1-2, F4-5 and F8, whereas the ER chaperones calnexin and calreticulin were distributed in all the fractions. Grp94 was also distributed in every fraction of C2C12 lysates, whereas it was concentrated in F1-2 and F7-8 of HeLa cells. Distribution of nNOS and SERCA2 differed widely between the two cell lines too, by accumulating in the non-buoyant and buoyant fractions, respectively, in HeLa cells, whereas they concentrated in medium-density fractions (F5-6) in C2C12 cells (Figure 2B and Figure S2). The majority of PMCA protein concentrated in buoyant fractions of both cell lines; however, only C2C12 cells showed the presence of detectable PMCA levels in F5-6 (Figure 2B).

The effect of Grp94 overexpression on protein distribution among ER vesicles was also investigated in a stably overexpressing-Grp94 C2C12 clone, previously characterized for cytoprotection and Ca^2+^ kinetics [9,10] (Figure 2C). In this clone, nNOS, PMCA, SERCA2 and Grp94 distribution mostly overlapped with that observed in C2C12 cells, showing an even greater concentration of PMCA and nNOS in the medium-density F5-7 fractions (Figure 2C and Figure S2).

### 2.3. Role of Grp94 Interactors in Antioxidant Cytoprotection

**Figure 3:**
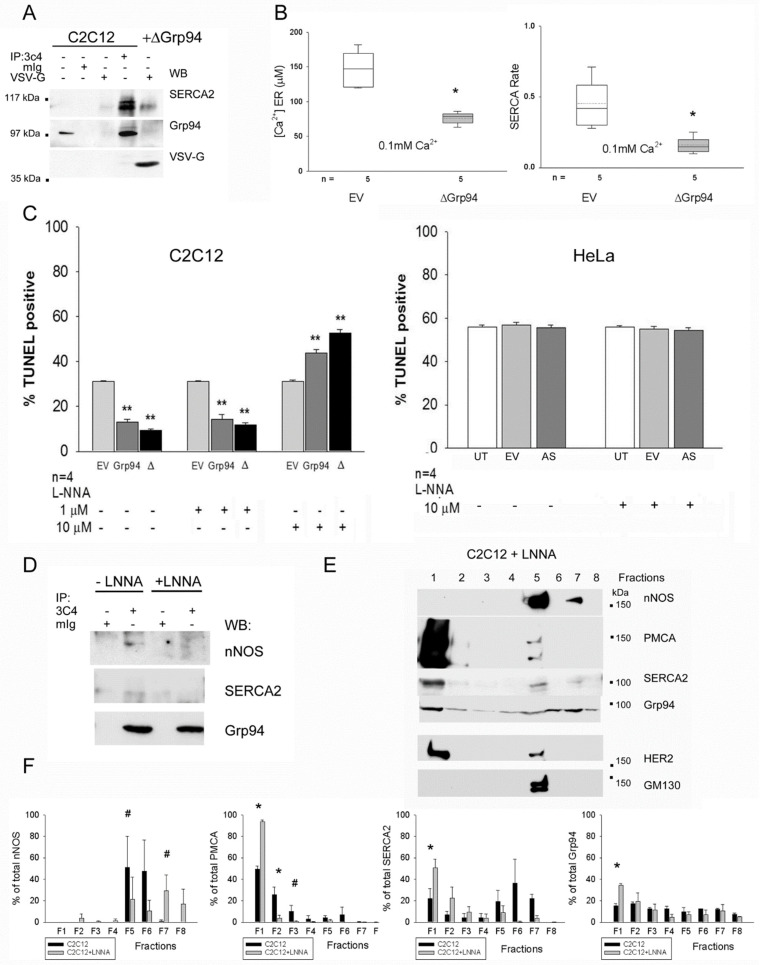
**SERCA2 and nNOS participate to Grp94-induced cytoprotection.** (**A**) Representative Western blots of lysates and immunoprecipitates from C2C12 cells, transfected or not with a construct expressing a deleted Grp94 (ΔGrp94). Immunoprecipitation was performed using either the anti-Grp94 3C4 antibody, or non-immune mouse G immunoglobulins (mIg) or anti-VSV-G tag antibodies and tested with anti-SERCA2, anti-Grp94 and anti-VSV-G antibodies. (**B**) ER Ca^2+^ levels and SERCA rate in C2C12 cells co-transfected with ER-AEQ probe and empty vector (EV) or ΔGrp94, after exposure to 0.1 mM Ca^2+^Cl_2_. n indicates the number of culture coverslips analyzed in at least two different experiments. * *p* ≤ 0.02. (**C**) Left panel: Histograms represent the percentage of TUNEL-positive nuclei detected after exposure to hydrogen peroxide of transiently transfected C2C12 cells with EV, Grp94 or ΔGrp94, treated (16 h) or not with different dosages of L-NNA. Values were calculated on the number of GFP-positive cells in cultures transfected with EV and Grp94, and on those positive for VSV-G tag immunofluorescence after transfection with ΔGrp94 (about 100 cells for each slide). *n* indicates the number of cultures analyzed from at least two different experiments. ANOVA ** *p* < 0.001. Right panel: Histograms represent the percentage of TUNEL-positive nuclei detected in HeLa cell cultures grown in standard conditions (UT), or transiently transfected with EV or antisense Grp94 cDNA (AS), after exposure to hydrogen peroxide, with (16 h) or without L-NNA. For transfected cultures, values were calculated on the number of positive cells for beta-galactosidase immunofluorescence (about 100 cells for each slide). n indicates the number of slides analyzed. (**D**) Representative Western blots of immunoprecipitates from C2C12 cells, with (16 h) or without 10 µM L-NNA, using the anti-Grp94 antibody 3C4 or mIg and labelling for nNOS, SERCA 2 and Grp94. Note the absence of nNOS and SERCA2 co-immunoprecipitation in the presence of L-NNA. (**E**) Representative Western blots of sucrose gradient fractionation of L-NNA exposed C2C12 cells stained for nNOS, PMCA, SERCA2 and Grp94. HER2 immunoreactivity was used as a plasma membrane marker and GM130 for Golgi. (**F**) Histograms illustrate the distribution of nNOS, PMCA, SERCA2 and Grp94 in C2C12 cells grown in the absence (black bar) or in the presence of L-NNA (grey bars). Values correspond to mean and SEM of 3 different experiments. Note the different distribution of SERCA2 and Grp94 after L-NNA exposure * *p* ≤ 0.03; # *p* = 0.07.

To clarify which one of these Grp94 interactions is primarily involved in cytoprotection and Ca^2+^ homeostasis of myogenic cells, we investigated the effects of a deleted form of Grp94 that does not interact with nNOS [7] (Figure 3). The VSV-G tagged Δ1-570 Grp94 polypeptide (ΔGrp94), corresponding to the dimerization domain and the C-terminus of the Grp94 molecule, was transiently expressed in C2C12 cells. Deleted Grp94 diffusely distributed within the ER and did not increase endogenous Grp94 protein levels (Figure S3). In these conditions, ΔGrp94 co-immunoprecipitated SERCA2, but not endogenous Grp94 (Figure 3A). ER Ca^2+^ measurements showed that ΔGrp94 expression significantly reduced ER Ca^2+^ content and delayed ER Ca^2+^ refilling rate, compared to what was observed in control, EV co-transfected cells (*p* < 0.01; Figure 3B), an effect similar to that caused by full-length Grp94-overexpression (Figure 1B, left). Exposure to hydrogen peroxide of ΔGrp94-transfected C2C12 cells induced a significantly decreased percentage of apoptosis, compared to that observed in control cells transfected with EV, and similar to what was found in full-length Grp94 overexpressing cells (Figure 3C). Altogether, these results strongly suggest the mechanistic involvement of SERCA2 in Grp94-induced cytoprotection.

To assess the contribution of nNOS, additional cytoprotection experiments were performed in the presence of Nω-Nitro-L-Arginine (L-NNA), a NOS inhibitor (Figure 3C, left). Firstly, we identified L-NNA dosages that do not reduce cell viability, either in basal conditions or after exposure to hydrogen peroxide (Figure S4). A large (30–60%), statistically significant increase in oxidant-induced apoptosis was observed in C2C12 cells overexpressing Grp94 or ΔGrp94 after exposure to 10 µM L-NNA (*p* < 0.01), whereas Grp94-cytoprotection was still maintained in parallel cultures exposed to a lower (1 µM) L-NNA dosage (*p* < 0.01; Figure 3C, left). Thus, the inhibition of an increasing number of nNOS molecules blunts Grp94 requirement for cytoprotection.

The possibility that nNOS activity would be independently required for cytoprotection was addressed by treating HeLa cells with the L-NNA inhibitor upon Grp94 silencing by cell transfection with antisense Grp94 cDNA (AS) (Figure 3C, right). Hydrogen peroxide exposure did not increase the percentage of apoptotic cells in any of the conditions tested, excluding the requirement of active nNOS in HeLa antioxidant protection, even in the presence of reduced Grp94 levels (Figure 3C, right).

Since both nNOS activity and SERCA2 interaction with Grp94 appear involved in C2C12 antioxidant cytoprotection, we explored whether L-NNA treatment affected Grp94 interactions with Ca^2+^-handling proteins. Exposure of C2C12 cells to the inhibitor abolished both nNOS and SERCA2 co-immunoprecipitation with Grp94 (Figure 3D) and significantly changed the pattern of SERCA2 and Grp94 distribution among ER vesicles (Figure 3E,F and Figure S5). Both proteins concentrated in light, buoyant fractions (F1) of L-NNA treated cells, compared to untreated ones (Student’s *t* test *p* < 0.01). The inhibitor also decreased the presence of PMCA and nNOS in the medium density fractions (F5–6), with the two proteins concentrated in buoyant (F1–2) and non-buoyant fractions (F7–8), respectively (Figure 3E,F and Figure S5).

### 2.4. Grp94 Protein-Interaction and Cytoprotection in Breast Cancer Cell Lines

To explore further the link between Grp94 overexpression, active nNOS and cytoprotection, we investigated breast cancer cells, which constitutively express high Grp94 protein levels. The human breast cancer cell lines MDA-MB-231 and HS578T were chosen because they do not overexpress the HER-2 receptor, to avoid the consequences on cell survival secondary to the loss of Grp94-mediated delivery of HER-2 molecules at plasmalemma [15].

**Figure 4:**
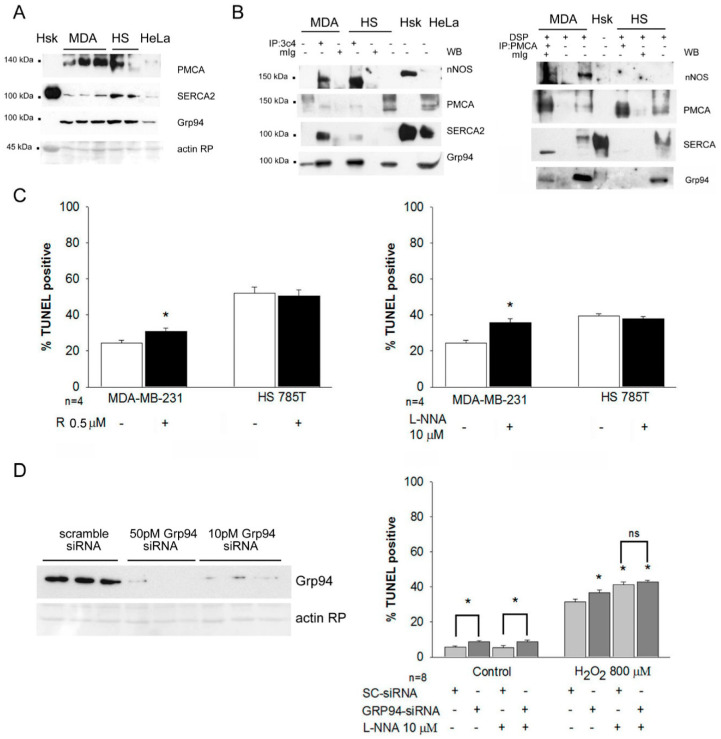
**Grp94 interaction with Ca^2+^-handling proteins and nNOS, and cytoprotection in breast cancer cell lines.** (**A**) Representative Western blots of whole homogenates from human skeletal muscle (Hsk) and of lysates of MDA-MB-231 (MDA), HS578T (HS) and HeLa cells tested with antibodies for PMCA, SERCA2 and Grp94. Red Ponceau staining of the blot at the level of actin was used for normalization. (**B**) Left panels: Representative Western blots of MDA and HS lysates (30 µg) and immunoprecipitates (IP, from 100–150 µg of lysate) using anti-Grp94 3C4 monoclonal antibody or non-immune mouse G immunoglobulins (mIg) tested for nNOS, PMCA and SERCA2 co-immunoprecipitation. Human skeletal muscle (Hsk) and HeLa cell lysates were loaded for reference. Right panels show reverse IP with anti-PMCA antibodies performed on lysates obtained after culture chemical cross-linking with DSP. (**C**) Histograms represent the percentage of TUNEL-positive nuclei of MDA-MB-231 and HS578T cells after exposure to hydrogen peroxide, with or without addition of radicicol (R) for 5 h (**A**) or L-NNA for 16 h (**B**) at the indicated dosages. Values were calculated on at least 100 cells for each slide. n indicates the number of slides analyzed. Student’s *t*-test, * *p* < 0.01. (**D**) Left panel: Representative Western blot of lysates of MDA-MB-231 cells 72 h after transfection with two different siRNA concentrations tested with antibodies for Grp94. Red Ponceau staining of the blot at the level of actin was used for normalization. Right panel: Histograms represent the percentage of TUNEL-positive nuclei detected in scramble (SC) and Grp94 siRNA transfected MDA-MB-231 cells, in growing conditions and after exposure to hydrogen peroxide, without or with addition of LNNA for the last 16 h at the indicated dosage. ANOVA, * *p* < 0.001.

Western blot analysis showed that Grp94 levels in MDA-MB-231 cells are similar to those observed in HeLa cells, while they appear almost doubled in HS578T cells (Figure 4A and Figure S6). Comparably, SERCA2 protein levels are similar in HeLa and MDA-MB-231 cells, whereas they are higher in HS578T cells. PMCA protein levels were upregulated in both breast cancer cell lines. IP experiments demonstrated comparable Grp94 interactions with nNOS and PMCA in both cancer cell lines, whereas that one with SERCA2 appeared more evident in MDA-MB-231 than in HS578T cells (Figure 4B). Reverse IP experiments, using PMCA antibodies and chemical cross-linking, confirmed the interaction with nNOS, SERCA2 and Grp94 in MDA-MB-231 lysates only (Figure 4B).

The two breast cancer lines were then exposed to hydrogen peroxide and the involvement of Grp94 and nNOS was evaluated by incubating parallel cultures with the non-selective Grp94 inhibitor radicicol [20], or L-NNA, respectively (Figure 4C). Apoptosis significantly increased in MDA-MB-231 cells after each treatment compared to what was observed with untreated cells (*p* < 0.01), whereas both inhibitors were ineffective in HS578T cells. Since the increase in apoptosis observed in MDA-MB-231 cells after the exposure to radicicol could involve inhibitory effects on the Grp94 paralog Hsp90, leading to nNOS degradation [21], similar experiments were performed in MDA-MB-231 cells after silencing of Grp94 mRNA and exposure to L-NNA (Figure 4D). Grp94 mRNA silencing significantly increased the percentage of apoptosis in basal conditions (*p* < 0.001) and after exposure to hydrogen peroxide (*p* < 0.04), compared to scramble siRNA-transfected cells (Figure 4D). The increase in apoptotic index showed by Grp94-silenced cells in basal conditions was not affected by L-NNA treatment (*p* = 0.002 vs. scramble-transfected cells). Conversely, the inhibitor significantly increased oxidant-induced apoptosis of both scramble- and Grp94 siRNA-transfected cells compared to untreated ones (*p* < 0.04), suggesting that nNOS-dependent antioxidant cytoprotection operates within the same Grp94-dependent pathway.

## 3. Discussion

This study demonstrates that Grp94-induced antioxidant cytoprotection does not come directly from the increased protein levels of the chaperone, but derives from the non-client interaction of Grp94 with Ca^2+^-handling proteins and active nNOS, by involving a specific ER vesicle population and influencing Ca^2+^ homeostasis.

The present findings add new relevant pieces of evidence concerning the controversy about Grp94 role in cell survival [12,13,14,15]. First, our results demonstrate that cytoprotection should not be expected as a general outcome from increased levels of this chaperone protein. As opposed to myogenic and neurogenic cell lines [8,9,10], a widely used epithelial cell line like HeLa did not show increased or reduced cytoprotection after Grp94 transient overexpression or downregulation, respectively. Consistently, breast cancer cells expressing high levels of Grp94 demonstrated a cell line-specific response in viability after Grp94 downregulation or inhibition ([15] and the present manuscript).

Secondly, we provide a new mechanism to explain the pleomorphism of the Grp94-dependent cytoprotective response. Lack of client-protein folding and maturation explains the loss of viability of Grp94-KO embryos or the requirement of serum for the growth of Grp94-KO embryonic fibroblasts [2]. In the case of non-client proteins, the loss of Grp94 jeopardizes the plasmalemmal localization of HER-2 receptors in HER-2-expressing breast cancer cells [15] or nNOS docking at the sub-sarcolemma of unloaded skeletal myofibers [7], inducing decreased cell viability and myofiber atrophy, respectively. Here we provide evidence that the interaction of Grp94 with two main Ca^2+^-handling proteins, SERCA2 and PMCA, and nNOS, is selectively involved in antioxidant cytoprotection of C2C12 myogenic cells and MDA-MB-231 breast cancer cells. In particular, Grp94 protein–protein interaction involved SERCA2, PMCA and nNOS, inducing a specific distribution of the proteins in a medium density population of ER vesicles, affecting Ca^2+^ homeostasis and resulting in cytoprotection.

Ca^2+^ measurements, by means of specifically targeted Ca^2+^ probes, revealed a significant decrease in ER Ca^2+^ content in transiently Grp94 overexpressing myogenic cells. The decrease in Ca^2+^ storage observed in C2C12 cells can be explained by the inhibitory effect played by Grp94 on SERCA2 activity, measured by the ER Ca^2+^ refilling rate, and confirmed by expressing ΔGrp94, which co-immunoprecipitated SERCA2 (Figure 3A), but not nNOS [7]. Other ER chaperones, such as calreticulin and ERp57 [22,23], have been described to interact with SERCA2 and to exert an inhibitory function. The data obtained with ΔGrp94 indicate that the Grp94-SERCA2 interaction involves the chaperone dimerization domain-C terminus, with which other proteins also interact [24]. Decreased ER Ca^2+^ levels justify the reduced percentage of oxidant-induced apoptosis. However, the Grp94 N-terminal domain also apparently plays a role in cytoprotection, since we showed here that exposure to the inhibitor radicicol, which binds close to the Grp94 nucleotide-binding domain, leads to increased oxidant-induced apoptosis of MDA-MB-231 cells.

However, HeLa cells did not show any change of ER Ca^2+^ content after Grp94 overexpression and showed Grp94-SERCA2 interactions together with calreticulin, suggesting their presence in the ER folding compartment. Indeed, Ca^2+^-imaging experiments in HeLa cells revealed that more than 70% of all Ca^2+^ puffs originated within a small perinuclear zone, where both Golgi apparatus and ER colocalize [25].

Ca^2+^ measurements revealed also a Grp94-dependent stimulatory effect on PMCA function, leading to increased rate of recovering basal cytosolic Ca^2+^ levels after stimulation, in C2C12 cells, but not in HeLa cells. In addition to extrude Ca^2+^, PMCA plays a structural function by binding nNOS through the PDZ domain. Binding to PMCA favors nNOS active state [26], but an active PMCA pump inhibits nNOS function [18]. Therefore, Grp94 interaction with and influence on PMCA would lead to opposite effects on nNOS activity. Results obtained by membrane fractionation suggest that non-plasmalemmal (inactive?) PMCA molecules are involved in Grp94-induced cytoprotection. Indeed, as opposed to HeLa cells, both control and Grp94-overexpressing C2C12 cells displayed SERCA2 and Grp94 accumulation in medium-density ER vesicle fractions, together with the majority of nNOS molecules and a minor proportion of PMCA molecules. The nature of this ER vesicle compartment remains to be determined. Its protein composition differs from the low-density buoyant fractions containing plasma membrane and related ER vesicles (the caveolae-enriched domain in [19]) and from Golgi-enriched high-density fraction, and appears strongly influenced by nNOS activity.

Stimulation of the soluble guanilate cyclase/cGMP/PKG pathway by low-level NO contributes to cytoprotective/antiapoptotic effects in many types of mammalian cells [27]. Using the NOS inhibitor L-NNA, here we show that nNOS activity is required for the antioxidant cytoprotection induced by Grp94 overexpression. In fact, a partial nNOS inhibition, to avoid collateral effects consequent to L-NNA high dosages, was effective in increasing oxidative stress-induced apoptosis only in those cell lines where multiple Grp94–protein interactions were detected, i.e., C2C12 and MDA-MB-231 cells. Indeed, C2C12 exposure to L-NNA disrupted Grp94 multiprotein interactions and their distribution among medium-density ER vesicles. Interestingly, Grp94 silencing combined with nNOS inhibition did not increase the degree of oxidant-induced apoptosis, supporting the hypothesis that Grp94 and nNOS act through the same mechanistic pathway leading to cytoprotection.

Whether active nNOS would be required upstream or downstream of Grp94 to achieve cytoprotection remains obscure. Independently from the soluble guanilate cyclase/cGMP/PKG pathway and in the presence of mild oxidation, NO can activate SERCA2 by means of pump S-glutationylation [28]; conversely, in the presence of high levels of oxidative stress, nitrosylation of SERCA2 tyrosine residues would inhibit the pump [29]. However, such a nNOS-mediated SERCA2 regulation would not be consistent with the effects observed after Grp94-overexpression in C2C12 cells, since S-glutationylation would antagonize the Grp94-induced SERCA2 inhibition and improve Ca^2+^ uptake within the ER lumen in basal conditions. Conversely, nitrotyrosination would enhance Grp94-induced SERCA2 inhibition during exposure to hydrogen peroxide and increase cytosolic Ca^2+^ load, which actually is delayed in Grp94-overexpressing C2C12 cells when exposed to an ionophore [9]. Relevantly, we report that this new cytoprotective mechanism is not restricted to myogenic cells. Grp94, PMCA and nNOS have been reported to be variably upregulated in cancer cells [14,26,30,31,32,33,34], whereas SERCA2 expression appears downregulated [32,35]. In particular, the PMCA2 isoform regulates viability of breast cancer cells by means of calcineurin and/or HER-2 receptor turnover [36,37], whereas PMCA1 appears to be involved in Ca^2+^ homeostasis [38]. Nitrotyrosine detection in cancer cells is a frequent observation and a negative prognostic marker [34,39]. NOS targeting has indeed been considered as an appealing strategy for cancer prevention and treatment [33,34,35,36,37,38,39,40,41]. The breast cancer cell line MDA-MB-231 (p53 mutant, HER-2 negative, estrogen receptor negative, progesterone receptor negative) is representative for high-malignancy and therapy-refractory breast cancer, and we show that oxidant-induced apoptosis can be increased more than 33% by either inhibiting nNOS or Grp94. Although L-NNA is not an isoform-specific competitive inhibitor, its affinity for nNOS is about 300-fold higher and its dissociation constant is lower than the corresponding ones for the inducible iNOS isoform [42], which also appears to be upregulated in several cancer cells [34,39]. We cannot exclude, however, that part of the increase in oxidant-induced apoptosis in the presence of L-NNA derives from iNOS inhibition.

In conclusion, the present findings indicate that Grp94-induced antioxidant cytoprotection involves interactions with Ca^2+^-handling proteins, SERCA2 and PMCA, and nNOS that, in turn, distribute within a distinct ER vesicle compartment and affect Ca^2+^ homeostasis. The disruption/disturbance of these interactions, by means of genetic or pharmacological interventions, appears promising to improve the killing of several cancers, among which are triple negative-p53 mutant breast cancers.

## 4. Material and Methods

### 4.1. Constructs

The constructs used to express full-length Grp94 for calcium studies (plasmid pBK-RSV94-TAG8) or TUNEL assay (pT94, which co-expresses GFP) were previously described [9]. Plasmid pVSV-G6B, which codifies for an N-terminal deleted Grp94 (AA 571–782) with a VSV-G tag was described in [7]. The corresponding empty vectors (EV) pBK-RSV-2.5X (Stratagene) and pT (Invitrogen) were used in control transfections. Grp94 silencing was obtained either using the As Grp94/βgal plasmid described in [10], or Grp94 siRNAs (ON-TARGET plus SMART pool siRNA HSP90B1, Dharmacon, GE Healthcare). EV (pBK-RSV expressing βgal) and scramble siRNA were used as control, respectively. For Ca^2+^ studies, the ER-AEQ and CYT-AEQ probes, selectively targeted to the ER and cytosol, respectively, were used [43,44].

### 4.2. Cell Culture and Transfection

Murine myogenic C2C12 cells and human epithelial HeLa cells were obtained from ECACC. The stably Grp94-overespressing C2C12 clone 8A11 was previously described [9]. The human breast cancer cell lines MDA-MB-231 and HS578T were provided by Sigma and by courtesy of Dr. Sbadzakay, respectively. All the cell lines were grown in proliferation medium (DMEM-high glucose containing 10% fetal calf serum, L-glutamine and antibiotics), except for HeLa cells, where standard DMEM was used. Transient transfections were performed using the calcium phosphate procedure, as described in [10]. Transfection with siRNA were performed using DharmaFECT transfection Reagents (GEHealthcare) following manufacturer instructions. About 15,000 cells/well were seeded in 24-well plates containing gelatin-coated glass slides and the day after were processed for transfection. Forty-eight hours after seeding, cells were used as described below.

### 4.3. Western Blotting and Immunoprecipitation

Whole cell lysates were prepared from 10 cm Petri dish subconfluent untransfected cultures or from 24-well transfected ones, and analyzed by SDS-PAGE and Western blot as previously described [10]. For IP studies, chemical cross-linking was performed on parallel cultures using the cell-permeant NHS-ester DSP (Pierce) as cross-linker, as previously described [7]. In brief, after adequate rinsing with phosphate-buffered saline (PBS), culture plates were exposed for 5 min at room temperature to 200 μg/mL DSP in PBS. Reaction was stopped by adding Tris 1 M pH 7.5. After rinse with cold PBS, cells were lysed with 1 mL of RIPA buffer added with anti-protease cocktail and proteins quantified using Bradford reagent (Sigma, Merck Life Science Srl, Milano, Italy). Cells treated with L-NNA (Sigma, Merck Life Science Srl, Milano, Italy) were lysed in the presence of the inhibitor. About 100 μg protein lysate was immunoprecipitated with 2 μg of either mouse monoclonal antibody anti-Grp94 clone 3c4 ([7] and Millipore Merck) or anti- PMCA clone 5F10 (Thermo Fisher Scientific, Monza, Italy). Mouse monoclonal anti-VSV-G clone P5D4 (Sigma, Merck Life Science Srl, Milano, Italy) was used for immunoprecipitation from C2C12 cells, in the presence and in the absence of transfection with plasmid pVSV-G6B. Non-immune mouse IgG or IgG1 (Sigma, Merck Life Science Srl, Milano, Italy) and IgG2a isotypes (Thermo Fisher Scientific, Monza, Italy) were used as controls. Immunocomplexes were pulled down after incubation with with 50 μL of anti-mouse Ig agarose-beads (Sigma) and centrifugation. Pellets were resuspended in gel loading buffer and boiled for 5 min in the presence of 5% β-mercaptoethanol, to release the immunoprecipitated proteins and to cleave the DSP linker, and were separated by SDS-PAGE. Anti n-NOS (BD Transduction Lab, Becton Dickinson & Co., Milano, Italy) and anti-SERCA2 (F1) (Santa Cruz Biotechnology, Heidelberg, Germany ) mouse monoclonal antibodies, and anti-calreticulin rabbit polyclonal antibodies (Stressgen) were used for Western blot, in addition to those used for IP.

### 4.4. Aequorin Ca^2+^ Measurements

Cells (0.5 × 10^5^) were plated on 13 mm coverslips and transfected after 24 h with either CYT- or ER-AEQ plasmids (0.5 µg) together with the indicated plasmids (1 µg), as described above. The day after transfection, cells were incubated at 37 °C with 5 µM native coelenterazine (Biotium) in mKRB (135 mM NaCl, 5 mM KCl, 1 mM MgCl_2_, 1 mM CaCl_2_, 20 mM HEPES, 11 mM glucose, pH 7.4 at 37 °C). Cells were transferred to the perfusion chamber and the experiments were performed in mKRB at 37 °C, collecting photons with a Photon counter (9125, Sens-Tech). ATP (10 or 100 μM) or histamine (100 μM) were added to Ca^2+^-free, EGTA (500 μM)-containing mKRB, together with 20 µM CPA, to avoid Ca^2+^ re-uptake in the ER. At the end of the experiments, cells were lysed with digitonin (100 µM) in a hypotonic, Ca^2+^-rich solution (10 mM CaCl_2_ in H_2_O) to consume the remaining unused aequorin pool and calibrate the experiment. Signal was converted into [Ca^2+^] as previously described [44]. For ER Ca^2+^ measurements and ER-AEQ reconstitution, luminal Ca^2+^ was reduced before coelenterazine addition by exposing the cells to CPA (20 µM) in Ca^2+^-free, EGTA (600 µM)-containing mKRB. After 1 h incubation at 4 °C, cells were extensively washed with Ca^2+^-free, EGTA (1 mM)-containing mKRB supplemented with bovine serum albumin (BSA, 2%). ER Ca^2+^ refilling was induced by cell perfusion with mKRB supplemented with CaCl_2_ (0.1 or 1 mM). SERCA rate was calculated as the angular coefficient of the slope ascending side of transients measured after ER-AEQ transfection. Ca^2+^ extrusion was calculated as the angular coefficient of the slope descending side of the rise induced by ATP cell stimulation, measured after CYT-AEQ transfection and normalized to the corresponding peak value.

### 4.5. Cytoprotection Experiments and TUNEL Assays

About 15,000–25,000 cells were seeded on gelatin-coated cells and exposed to 800 μM H_2_O_2_ in 0.5 mL/well of growth medium for 5 h, based on previous experience on C2C12 cells [10] and preliminary testing of dosage effects on apoptosis of HeLa cells (Figure S7).

A 10 mM aqueous solution of L-NNA was prepared and stored in aliquots at −20 °C. Radicicol (Sigma) was dissolved in ethanol (5 mM) and added to culture medium at a final concentration of 0.5 μM [20]. Either inhibitor was added to culture about 16 h before the oxidative challenge.

Nucleosomal DNA fragmentation was visualized by TUNEL assay. Cells were fixed and incubated with 80U TdT (Roche) and 50 pmol of Tetramethyl-rhodamine-5dUTP (ENZO Italy) following manufacturer instructions. After rinsing with PBS, autofluorescence was quenched by incubation for 15 min at RT with 50 mM ammonium chloride and coverslips mounted on glass slides with buffered glycerol added with 2 μg/mL of 4,8-diamine-2-phenylindol (DAPI; Sigma) and examined using an Axioplan epifluorescence microscope (Zeiss, Arese, Italy).

Except for siRNA treated cells, transfected cells were identified by means of GFP co-expression, or by immunofluorescence for VSV-G or β-gal, performed as previously described [9,10], using an Alexa 488-conjugated secondary antibody (Invitrogen).

About 100–150 transfected cells were evaluated for each cover slip; each experiment was performed in triplicate; values correspond to at least two independent experiments. Evaluation of cell necrosis was performed on coverslips in parallel wells by adding propidium iodide (PI) to the incubation medium as described in [9].

### 4.6. Subcellular Fractionation

About 5 to 6–10 cm Petri dishes of subconfluent cultures were rinsed thrice with PBS, scraped and lysed in 2 mL of ice cold 0.5 M sodium carbonate using 50 strokes of a Dounce glass homogenizer on ice. As described by [19], lysate was added to 2 mL 90% sucrose in 25 mM Mes pH 6.5, 150 mM NaCl, 5 mM EDTA and 1 mM PMSF. This solution was overlayed with 38% and 5% sucrose in 0.25 M sodium carbonate to form a discontinuous gradient and centrifuged overnight at 4C at 33,000 rev/min in a SW41 rotor using a Beckman L7 Ultracentrifuge. For cells treated with L-NNA, the inhibitor was added to both lysis buffer and sucrose gradient. The first 2 mL at the top of the gradient were discarded and eight 1 mL fractions collected in plastic tubes. Protein was precipitated and sucrose removed by chlorophorm/phenol extraction [45]. In brief, 4 mL methanol, 1 mL chlorophorm and 3 mL H_2_O were added to each 1 mL fraction, thoroughly mixing after each addition and then centrifuging at 11,000× *g* for 10 min. After removal of the aqueous upper layer, 4.3 mL of methanol was added, the tube was inverted three times and centrifuged again at 11,000× *g* for 10 min. Pellets were suspended in 100 µL of electrophoresis buffer and about 2/3 or all of the volume was loaded either in 7.5% or 6–12% gradient reducing SDS-PAGE. Western blots were stained as described above, using also anti-HER-2, anti-TOM20 and anti-calnexin antibodies (Santa Cruz Biotechnology), and anti-GM130 antibody (BD Transduction lab) to map fraction content.

About 3–4 fractionation experiments were done for each cell line/condition. Protein percentage in each fraction was calculated after densitometry on the total value corresponding to eight fractions.

### 4.7. Statistical Analyses

Data are expressed as mean and SEM. ANOVA was used to evaluate statistically significant differences among groups followed by Newman–Keuls post hoc test. Unpaired Student’s *t*-test was used when comparing two groups. *p* values less than or equal to 0.05 were considered statistically significant. Analyses were performed using Statistical Package SigmaStat version 2.0 (Systat Software GmbH, Dusseldorf, Germany).

## Supplementary Materials

The following are available online at https://www.mdpi.com/article/10.3390/ijms23062915/s1.
