# Supplementary material for: Active nNOS Is Required for Grp94-Induced Antioxidant Cytoprotection: A Lesson from Myogenic to Cancer Cells
Source: Int J Mol Sci. 2022 Mar 8;23(6):2915. doi: 10.3390/ijms23062915 (PMC8954037; doi:10.3390/ijms23062915)
Supplement: Supplementary file 1 [file ijms-23-02915-s001.zip › ijms-1570404-supplementary.pdf]

Supplemental Figures

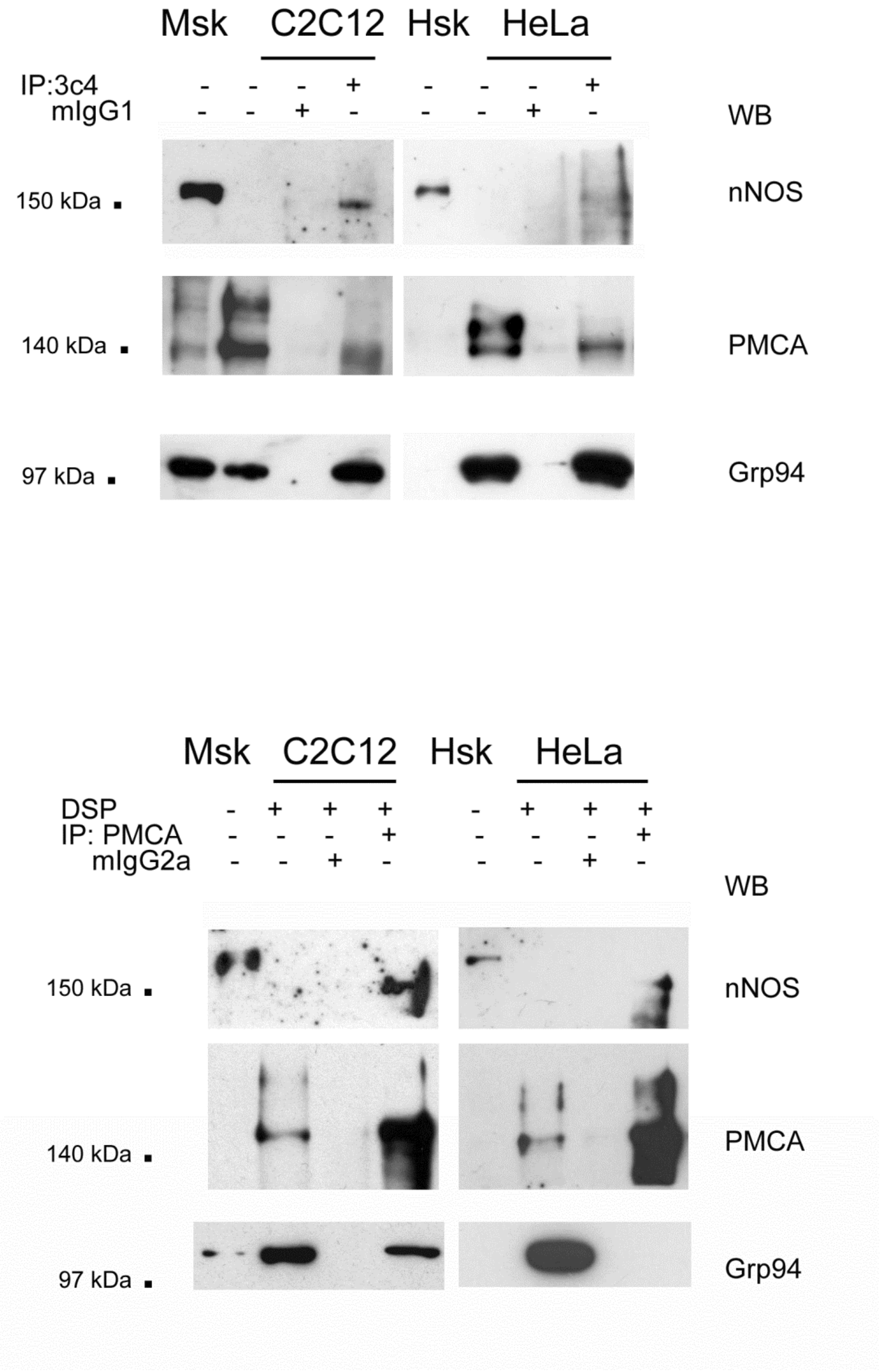

Figure S1. Co-immunoprecipitation analyses on C2C12 and HeLa cells with anti-Grp94 3c4 and anti-PMCA antibodies using isotype IgG as controls.

Msk= mouse skeletal muscle; Hsk: human skeletal muscle. The nNOS μ-isoform detectable in skeletal muscle displays a higher molecular weight than the more widespread α-isoform.

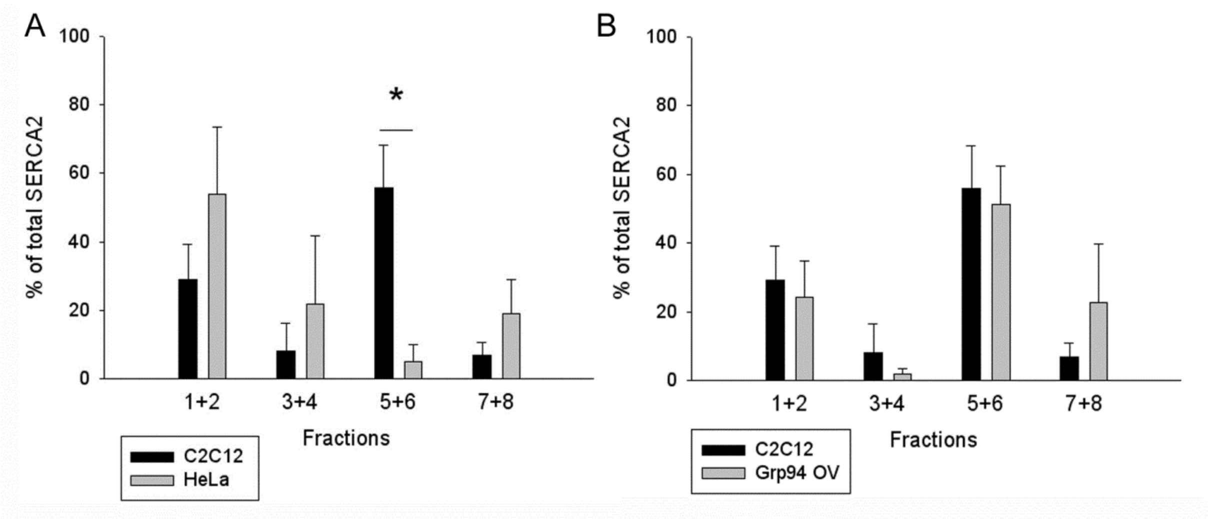

Figure S2. Histograms of SERCA2 distribution in pooled sucrose gradient fractions of C2C12, HeLa and Grp94 overexpressing (OV) C2C12 clone cells.

*P=0.01, n=3

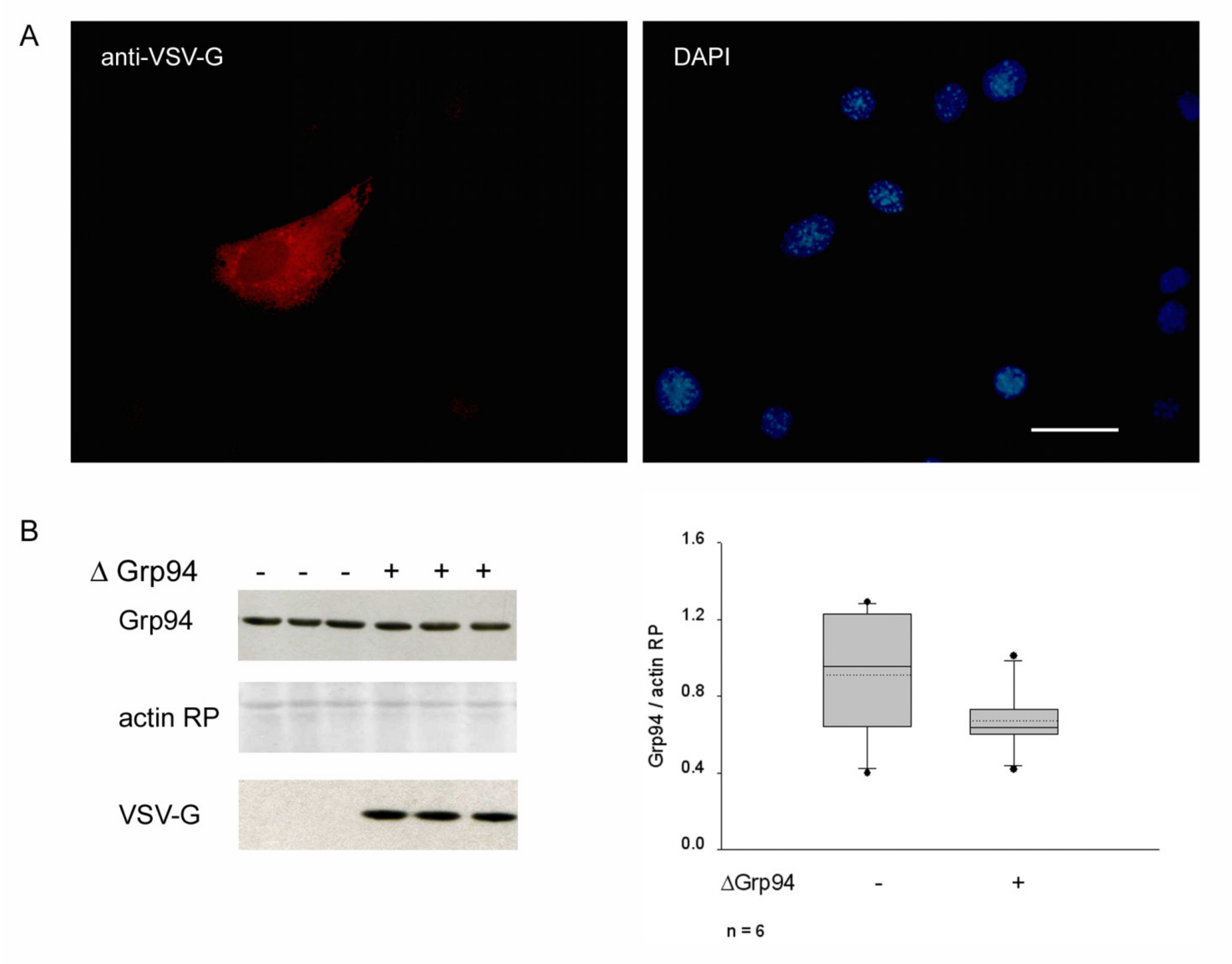

Figure S3: **Distribution of Δ1-570 Grp94 and effect on endogenous Grp94 protein levels in C2C12 cells.**

A) Indirect immunofluorescence for VSV-G-tag identifying Δ1-570 Grp94 protein in C2C12 cells 24h after transfection with pVSV-G 6B. Slides were counterstained with DAPI to visualize nuclei. Bar=30μm

B) Representative Western blots and box plots showing Grp94 levels in control and Δ Grp94 transfected C2C12 cells. Grp94 densitometric values were normalized to actin staining with Red Ponceau (RP). VSV-G staining demonstrates the presence of Δ Grp94.

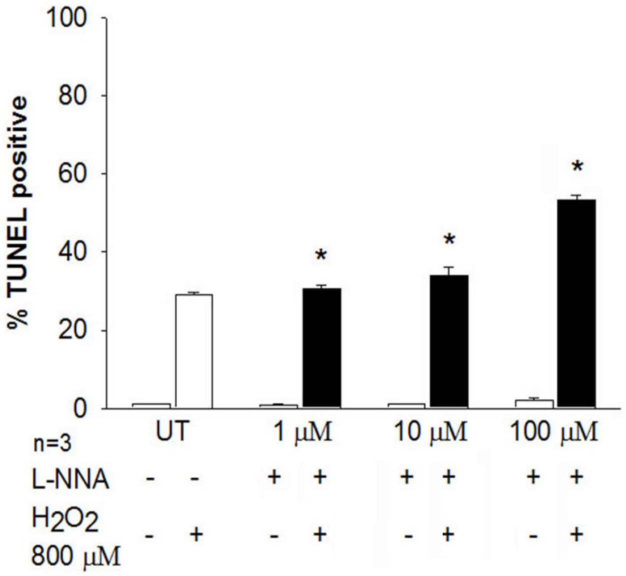

Figure S4: **Effects of L-NNA dosage on apoptosis of C2C12 cells in basal conditions and after exposure to hydrogen peroxide.**

Histograms represent the percentage of TUNEL-positive nuclei detected in C2C12 cultures, in standard growth conditions (white bars) or after exposure to hydrogen peroxide (black bars), in the absence (UT) or in the presence (16 h) of the indicated dosage of L-NNA. n indicates the number of slides examined, values were calculated on more than 100 cells for each slide. ANOVA * $P<0.001$. Note the absence of effect on cell mortality of the inhibitor in standard growth conditions.

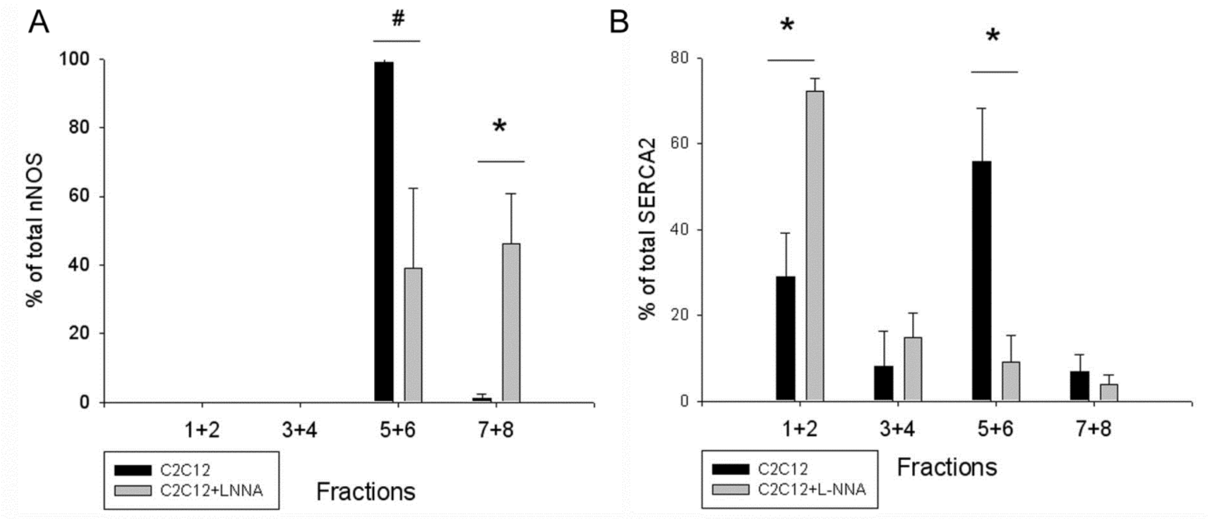

Figure S5: Histograms of nNOS and SERCA2 distribution in pooled sucrose gradient fractions of C2C12 cells with or without L-NNA.

*P≤0.03; # P=0.06; n=3

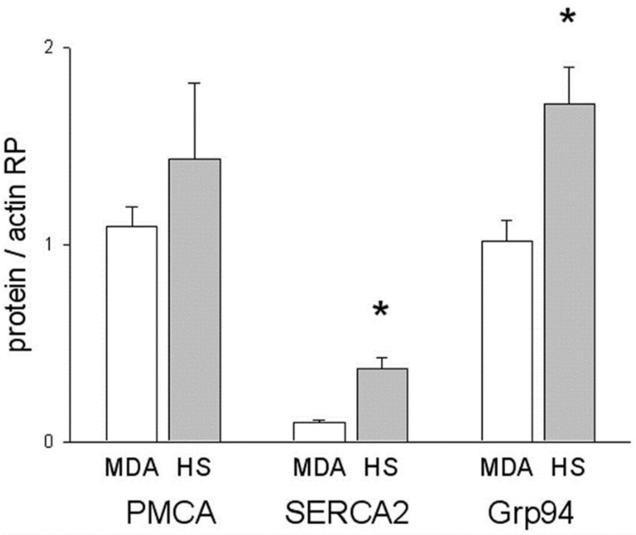

Figure S6: **PMCA, SERCA2 and Grp94 protein levels in MDA-MB-231 (MDA) and HS578T (HS) cells.**

Histograms of relative PMCA, SERCA2 and Grp94 protein levels observed in MDA-MB-231 (MDA) and HS578T (HS) cells (normalized for actin signals). Data were collected from 3 different cultures for each cell line. Student's t test * $P \leq 0.03$.

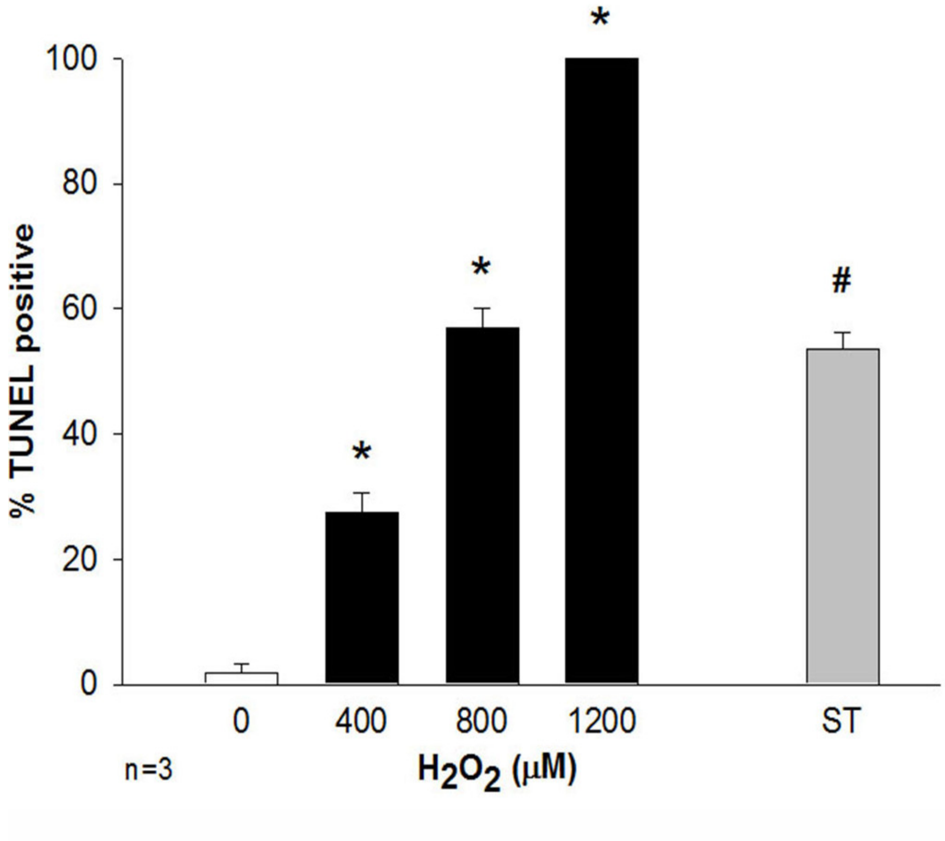

Figure S7: **Effects of different concentrations of hydrogen peroxide on HeLa cell apoptosis.**

Histograms of the percentage of TUNEL positive nuclei of HeLa cells, detected after exposure to different concentrations of hydrogen peroxide for 5 h. n indicates the number of slides examined for each experimental condition in two different experiments. ST indicate apoptosis observed in parallel cultures exposed to 1µM staurosporine for 3.5 h. ANOVA,* P<0.001; Student's t-test, # P<0.001 vs 0.
